# Is biofilm removal properly assessed? Comparison of different quantification methods in a 96-well plate system

**DOI:** 10.1007/s00253-016-7396-9

**Published:** 2016-02-29

**Authors:** Philipp Stiefel, Urs Rosenberg, Jana Schneider, Stefan Mauerhofer, Katharina Maniura-Weber, Qun Ren

**Affiliations:** Laboratory for Biointerfaces, Empa, Swiss Federal Laboratories for Materials Science and Technology, Lerchenfeldstrasse 5, CH-9014 St. Gallen, Switzerland; Borer Chemie AG, Gewerbestrasse 13, 4528 Zuchwil, Switzerland

**Keywords:** Biofilm quantification, Biofilm removal, Microtiter plate, Enzymatic cleaner, Method comparison

## Abstract

Various methods have been reported to quantify total biofilm or different components of biofilm; however, these methods are often confusedly used, leading to discrepancies and misleading results. In this study, different methods for quantification of biofilm, including those for total biomass, total amount of bacterial cells, viable cell number, and amount of extracellular polymeric substances, were systematically compared in microtiter plates. To evaluate which method is suitable for assessment of biofilm removal and for bacterial killing, biofilm samples were treated with various cleaners possessing removing and/or killing capacities. It was found that most of the methods tested in this study in general exhibited high reproducibility and repeatability. Crystal Violet staining was a simple but reliable method for total biomass quantification. Total bacteria cell numbers could be reliably quantified by the fluorescent DNA-binding dye Acridine Orange. Viable cells could be quantified by either an ATP-based assay or a proliferation assay. Both of these viability methods showed a broad detection range and led to precise measurement. For quantification of proteins in the biofilm, staining with fluorescein isothiocyanate was most suitable. Furthermore, it was revealed that a combination of different methods is required to determine if a cleaner kills or removes biofilm.

**Electronic supplementary material:**

The online version of this article (doi:10.1007/s00253-016-7396-9) contains supplementary material, which is available to authorized users.

## Introduction

Under natural conditions, most bacteria occur in the form of a biofilm. They adhere to surfaces embedded in a self-produced layer of extracellular polymeric substances (EPS) (Flemming and Wingender 2001; Sutherland 2001). The EPS protect bacteria against environmental influences such as UV irradiation, antibiotics, and disinfection, which make them much more tolerant to these influences compared to planktonic bacterial cells (Cochran et al. 2000; Elasri and Miller 1999; Stewart and Costerton 2001). The unique structure of biofilm makes it difficult to be removed. Particular precautions have to be taken in the field of health care. Hospital-acquired infections, which often arise from incomplete removal of biofilm from instruments and device surfaces, account for a substantial part of health problems and costs (Zimlichman et al. 2013).

An example is the transfer of pathogens on flexible endoscopes. Endoscopes get in contact with different body fluids and provide ideal surfaces for biofilm formation. Viable bacteria were isolated from many endoscope channels even after the cleaning and disinfection process (Alfa et al. 1999; Chiu et al. 2012; Kovaleva et al. 2013; Pajkos et al. 2004). Thus, biofilm cleaners need to be carefully developed and evaluated for their performance. Different methods have been reported for biofilm analysis. Viable bacteria can be detected by monitoring their metabolic activity (e.g., ATP by BacTiter-Glo™ assay (Berney et al. 2008) or respiratory electrons by tetrazolium salt (Hatzinger et al. 2003)), membrane integrity (e.g., live/dead staining by SYTO9 and propidium iodide (Tawakoli et al. 2013)), or ability to grow (e.g., colony forming units or time to regrow to a specific turbidity (Alt et al. 2004)). The total amount of live and dead bacteria can be measured using DNA-binding dyes (e.g., DAPI, Hoechst, SYTO9 or Acridine Orange (Peeters et al. 2008; Palestrant et al. 2004)) or qPCR. Also, the EPS compounds can be visualized and quantified. Proteins can be stained by specific dyes (e.g., SYPRO Ruby, CBQCA, or NanoOrange) or detected by specific reactions (e.g., Lowry method). The same is possible for polysaccharides which can, for example, be stained by fluorescently labeled lectins (e.g., ConA-FITC (Chen et al. 2007) binds α-d-mannopyranosyl and α-d-glucopyranosyl residues such as in amylopectin and dextran) and other dyes (e.g., Calcofluor White (Chen et al. 2007) binds β(1→4) linked d-glucose or derivatives such as cellulose and chitin) or detected by the phenol sulfuric acid method (Dubois et al. 1951). Fluorescein isothiocyanate (FITC) is also supposed to label proteins by reaction of the isothiocyanate group with primary and secondary amine groups to form a covalent bonding (Chen et al. 2007). Many of these methods are not yet described for biofilm quantification in a microtiter plate screening system. Simpler staining methods such as Crystal Violet (O’Toole 2011; Stepanovic et al. 2000) and Safranin Red (Patterson et al. 2010) which target total biomass were more frequently used. These dyes bind to negative charges and therefore target many different molecules of bacteria and EPS. An advantage of these methods is the simplicity and the direct optical visualization. However, the question remains open how quantitative and reliable these methods are in comparison to each other. Advantages and disadvantages of different methods were discussed (Pantanella et al. 2013), but experimental data were rarely used to support the statements (Peeters et al. 2008). Furthermore, only few quantification methods were compared for biofilm removal studies (Pitts et al. 2003). Especially, when biofilm removal or disinfection is concerned, little is discussed about which method is suitable for which purpose and what the detection limits are. Dependent on the used method, the readout will vary. If a cleaner kills the bacteria rather than removes them, the outcome for the efficiency will differ if a quantification method is selected to determine total biofilm or measure bacterial viability. In reported studies, conclusions for cleaning efficiency were often drawn based on detection of viable cells and not on actual biofilm removal (Hadi et al. 2010; Vickery et al. 2004). Thus, there is a need to conduct the experiments under the same conditions and compare different methods systematically.

In this study, biofilm formed in microplates and cleaners having biofilm-removing and/or bacteria-killing ability were used to investigate different biofilm quantification methods in 96-well plates. These include methods to determine total biomass, total bacteria, viable bacteria, and EPS including proteins and polysaccharides. As model organisms, the clinically relevant Gram-negative *Pseudomonas aeruginosa* and Gram-positive *Staphylococcus aureus* were employed (Pendleton et al. 2013). The cleaning efficiency of a novel enzymatic cleaner was compared with that of five commercial products. The advantages and disadvantages of the different methods are elaborated in detail in this report. The results and findings obtained here not only are scientifically interesting but also more importantly will allow correct assessment and monitoring of medical and environmental products, e.g., endoscope cleaners and disinfectants, for their efficacy in biofilm removal and/or killing bacterial cells.

## Materials and methods

### Chemicals and reagents

Chemicals and reagents used for bacteria growth, cleaner formulation, and biofilm detection were purchased from Sigma-Aldrich (Switzerland) if not mentioned elsewise.

### Bacterial strains and cultivation conditions

Bacterial strains were obtained from the Leibniz Institute DSMZ—German Collection of Microorganisms and Cell Cultures. *Pseudomonas aeruginosa* (DSM No. 1117) and *Staphylococcus aureus* (DSM No. 20231) were grown on tryptic soy agar at 37 °C. Liquid cultures were grown in 30 % tryptic soy broth (TSB, 9 g/l which corresponds to 30 % of recommended concentration) supplemented with 2.5 g/l glucose at 37 °C and 160 rpm.

### Biofilm formation

Overnight cultures were diluted to OD_600 nm_ of 0.2 in 30 % TSB supplemented with 2.5 g/l glucose. Two hundred microliters of bacteria suspension per well were added to transparent (for absorbance), black (for fluorescence), or white (for luminescence) flat-bottom polystyrene 96-well plates (BRAND*plates*® pureGrade™). Samples were arranged as indicated in Figure S1a. To determine the staining background, two rows of the microplate were filled with medium without bacteria. One column was left empty (no medium and bacteria) during biofilm formation and later was also washed and treated with 0.9 % NaCl solution to determine the staining background signals of the plate. Plates were incubated for 24 h at 33 °C and 40 rpm. The biofilm formed in the wells was washed once with 350 μl 0.9 % NaCl solution before the treatment with cleaner.

### Cleaner treatment

In this study, five commercially available high-end endoscope cleaners (cleaners A–E) and one newly developed cleaner X were chosen, all of which contain enzymes and/or claim to be efficient in removing biofilm (Table 1).

**Table 1:** Summary of biofilm removal capacity of the cleaners tested in this study

Cleaner	Enzymes supplemented in the cleaners	Claim for biofilm removal	Biomass		Amount of bacteria		Amount of viable bacteria		Amount of EPS	
			P.a.	S.a.	P.a.	S.a.	P.a.	S.a.	P.a.	S.a.
A	Protease, lipase, amylase	Yes	++	+++	++	+++	+++	++	++	+++
B	Protease, amylase, cellulase	Yes	+++	+++	+++	+++	+++	++	+++	+++
C	Protease, lipase, amylase	None	++	+	0	0	+++	+++	0	0
D	Protease, lipase, amylase, cellulase, mannanase	Yes	0	++	0	++	+	+++	0	+++
E	None	Yes	+	0	0	0	++	+++	0	++
X	4 enzymes	Yes	+++	+++	+++	+++	+++	+++	+++	+++

P.a., *Pseudomonas aeruginosa*; S.a., *Staphylococcus aureus*; +++, strong (>80 % in average of the used methods or >90 % in one of the methods); ++, medium (>50 % in average, but none >90 %); +, weak (25–50 % in average); and 0, no biofilm reduction (<25 % in average). Those terms were applied to all methods, except for the viability of S.a. where the threshold for +++ was set to 99 % due to strong reduction of all cleaners. More details on percent reduction of individual methods are summarized in Table S1

All cleaners were used at a concentration of 1 % (as recommended by the manufacturers) in freshly prepared water of standardized hardness (WSH) containing 1.25 mM MgCl_2_, 2.5 mM CaCl_2_, and 3.33 mM NaHCO_3_ in deionized water. The samples were arranged as described in Figure S1b. Each column (six wells with bacteria, two wells with medium only) was treated with a different cleaner. A mixture of 1 % SDS, 1 % EDTA, 1 % NaOH, and 0.1 % NaClO was used as the positive control, and 0.9 % NaCl solution was used as the negative control. Treatment was done with 250 μl cleaner per well for 40 min at 25 °C. Two columns for background control were also treated with 0.9 % NaCl solution: one to determine the background of cells without staining and the other one for the staining background of the plate (the empty one without bacteria and medium). Before applying the different detection methods, the wells were washed three times with 350 μl 0.9 % NaCl solution to remove the cleaner and dislodged debris. Finally, all liquid was removed before immediately applying the specific quantification method.

### Biofilm quantification

After the application of the quantification methods described below, absorbance, fluorescence, or luminescence were measured with a Synergy HT Multi-Detection Microplate Reader (BioTek®).

#### Total Biomass

The classical dyes such as Crystal Violet and Safranin Red bind to negatively charged molecules and can be used to stain and quantify total biomass comprising bacteria and EPS.

##### Crystal Violet staining

Two hundred fifty microliters of 0.5 % Crystal Violet (CV) was added per well (except one column which was used to determine the background of the cells and medium). The plate was incubated for 30 min at 25 °C before removing the staining solution, and then washed three times with 350 μl 0.9 % NaCl solution. After removing the washing solution, 100 μl 96 % EtOH was added per well to dissolve the biofilm-bound CV by gently knocking the plate. Absorbance was measured at 595 nm.

##### Safranin Red staining

Safranin Red staining was similar to CV staining, except that 0.5 % Safranin Red was used and absorbance was measured at 535 nm.

##### Congo Red staining

Congo Red staining was similar to CV staining, except that 0.5 % Congo Red was used and absorbance was measured at 500 nm.

#### Total amount of bacterial cells

Dyes binding to DNA and RNA such as SYTO9 and Acridine Orange can be used to quantify both live and dead bacterial cells and provide an insight into the total amount of bacterial cells in the biofilm.

##### SYTO9 staining

One hundred microliters of 2.5 μM SYTO9 (Life Technologies) in 0.9 % NaCl solution was added per well (except one column which was used to determine the background of the cells and medium). The plate was closed with sealing aluminum foil (AlumaSeal II™, Carl Roth) before vortexing for 10 min to detach biofilm and incubated for additional 5 min. Fluorescence intensity was measured using excitation filter 485/20 nm and emission filter 528/20 nm (gain 70).

##### Acridine Orange staining

Acridine Orange solution (2 % in H_2_O) was diluted 1:100 in Walpole’s buffer (27.2 g/l sodium acetate trihydrate, adjusted to pH 4 with glacial acetic acid). Two hundred fifty microliters of the diluted Acridine Orange was added per well. After 15 min incubation in the dark, the staining solution was removed and the plate was washed three times with 350 μl 0.9 % NaCl solution. After removing the washing solution, 100 μl 0.9 % NaCl solution was added and the plate was closed with sealing aluminum foil before vortexing for 10 min. Fluorescence intensity using excitation filter 485/20 nm and emission filter 528/20 nm (gain 50) was measured.

#### Amount of living bacterial cells

Methods which measure typical parameters of living bacteria such as metabolic activity, membrane integrity, or proliferation can be used to specifically quantify these bacterial cells.

##### BacTiter-Glo assay

One hundred microliters of TSB was added to each well, and the plate was closed with sealing aluminum foil. The plate was vortexed for 10 min to detach the bacteria. One hundred microliters of BacTiter-Glo reagent (BacTiter-Glo™ Microbial Cell Viability Assay, Promega) prepared according to the manufacturer’s instruction was added per well. The plates were incubated for 5 min in the dark on an orbital shaker. The luminescence intensity was measured with gain 135 for 1 s per well.

##### Turbidity threshold method

Two hundred fifty microliters of 30 % TSB (9 g/l) was added per well after cleaner treatment, and the plate was closed with a sealing transparent foil (Breathe-Easy, Carl Roth). The plate was incubated in the microplate reader at 33 °C with medium shaking (18 Hz—the preset value of the instrument). Optical density at 600 nm was measured every 30 min for 24 h. A dilution series of a bacterial culture enabled a correlation between cell numbers and time needed to reach a desired optical density (OD).

##### Tetrazolium salt assay

Two hundred microliters of 30 % TSB containing 0.5 mg/ml 2-(4-iodophenyl)-3-(4-nitrophenyl)-5-phenyl-2*H*-tetrazolium chloride (INT) was added to each well. The plate was incubated for 2 h at 33 °C with shaking at 40 rpm. The medium was removed, and 200 ml dimethyl sulfoxide (DMSO) was added to dissolve the dye from the biofilm by knocking the plate carefully. Absorbance was measured at 470 nm.

##### SYTO9/PI staining

Continuing from SYTO9 staining described above, 100 μl of 15 μM propidium iodide in 0.9 % NaCl solution was added. After 15 min incubation, fluorescence intensities using excitation filter 485/20 nm and emission filters 528/20 and 645/20 nm (gain 70) were measured.

#### Biofilm EPS - amount of proteins

##### FITC staining

Two hundred fifty microliters of FITC solution (20 μg/ml fluorescein isothiocyanate in ddH_2_O prepared from 10 mg/ml stock in EtOH) was added to each well. After 30 min incubation in the dark, the staining solution was removed and the plate was washed twice with 350 μl 0.9 % NaCl solution. After removing the washing solution, 100 μl ddH_2_O was added and the plate was closed with sealing aluminum foil before vortexing for 10 min. Fluorescence intensity using excitation filter 485/20 nm and emission filter 528/20 nm (gain 70) was measured.

##### SYPRO Ruby staining

SYPRO Ruby staining is similar to FITC staining, except that undiluted SYPRO Ruby solution (FilmTracer™ SYPRO® Ruby Biofilm Matrix Stain, Life Technologies) was used for 15 min and fluorescence intensity was measured using excitation filter 460/40 nm and emission filter 645/40 nm (gain 80).

##### BCA/NanoOrange/CBQCA assays

NanoOrange® Protein Quantitation Kit (Life Technologies), CBQCA Protein Quantitation Kit (Life Technologies), and Micro BCA™ Protein Assay Kit (Life Technologies) were performed according to manufacturers’ instructions.

##### Lowry assay

Two hundred fifty microliters of complex-forming reagent (100:1:1 (*v*:*v*:*v*) mixture of 2 % sodium carbonate, 1 % copper(II) sulfate pentahydrate, and 2 % sodium potassium tartrate tetrahydrate in ddH_2_O) was added per well. The plate was incubated for 10 min at 25 °C. Fifty microliters of Folin & Ciocalteu’s phenol reagent was added. The plate was incubated for 30 min at 25 °C before measuring absorbance at 540, 650, and 750 nm.

#### Biofilm EPS - amount of polysaccharides

##### Calcofluor White staining

Two hundred fifty microliters of Calcofluor White solution (1 mg/ml in dH_2_0) was added per well and incubated for 15 min in the dark. The staining solution was removed, and the plate was washed twice with 350 μl 0.9 % NaCl solution. After removing the washing solution, 200 μl 96 % EtOH was added per well to dissolve the biofilm-bound Calcofluor White by gently knocking the plate. Fluorescence intensity using excitation filter 360/40 nm and emission filter 460/40 nm (gain 80) was measured.

##### ConA-FITC staining

Two hundred fifty microliters of 50 μg/ml ConA-FITC was added per well (diluted in 0.9 % NaCl from 5 mg/ml stock in 0.1 M NaHCO_3_). After 15 min incubation in the dark, the staining solution was removed and the plate was washed twice with 350 μl 0.9 % NaCl solution. After removing the washing solution, 100 μl 0.9 % NaCl solution was added and the plate was closed with sealing aluminum foil before vortexing for 10 min. Fluorescence intensity using excitation filter 485/20 nm and emission filter 528/20 nm (gain 80) was measured.

### Statistical analysis

For each sample, the biofilm value was calculated by subtracting the mean value of the two wells with only medium from the arithmetic mean of six wells with biofilm. Sample standard deviations were calculated from the values of the six similarly treated wells. The statistical significance was determined for each data set using the unpaired, parametric, two-tailed Student’s *t* test. The value of the negative control (0.9 % NaCl treated) was set to 100 % and the other values calculated accordingly. Three independent experiments with six repeats per condition in each experiment were performed for each detection method. Individual repeats displayed the same trend for all detection methods.

In this study a minimal reliable signal detected is defined using the IUPAC definition (http://goldbook.iupac.org/L03540.html, IUPAC Compendium of Chemical Terminology - the Gold Book) to enable clear discrimination from background noise: the signal should be larger than the background signal by three times the standard deviation of the background. Maximal detectable reduction (MDR) values were calculated using the following formula:

$$ MDR\kern0.5em \left[\%\right]=100-\frac{MS-BG}{SNC-BG}\times 100=100-\frac{3*SD}{SNC-BG}\times 100 $$

- BG, background signal of stained wells which did not contain bacteria and medium
- SD, standard deviation of the BG
- MS, minimal reliable signal (= BG + 3*SD)
- SNC, signal of negative control wells containing bacteria treated with 0.9 % NaCl solution instead of a cleaner

## Results

### Biofilm formation and treatment

Different conditions such as incubation temperature and duration were investigated for biofilm formation in 96-well plates. It was found that incubation at 33 °C and 40 rpm for 24 h was sufficient to obtain an adequate amount of biofilm for subsequent quantification. Prolonged incubation for 48 h and/or incubation at 37 °C did not lead to a significantly higher amount of biofilm (less than 10 % in average). It was observed that *P. aeruginosa* formed the biofilm mainly at the liquid/air interface on the walls (Fig. S2a), whereas the *S. aureus* biofilm mainly adhered to the bottom and in the corners (Fig. S2b). This observation has been reported previously (O’Toole 2011). However, the different locations of biofilm should not influence the cleaning process as static condition was used and the biofilm was completely covered with the cleaner. Room temperature (25 °C) was selected for biofilm treatment with cleaners as this temperature is most often used for manually applied cleaning solutions. Incubation for 40 min was chosen for treatment as it was found to give reproducible results.

### Detection methods

Biofilm in 96-well plates treated with different solutions (see “Materials and methods”) was used to investigate the suitability and detection limits of various biofilm detection methods. The maximal detectable reduction (MDR) was used to reflect the sensitivity and the detection range of a method as described in “Materials and methods.”

#### Total biomass

**Fig. 1:**
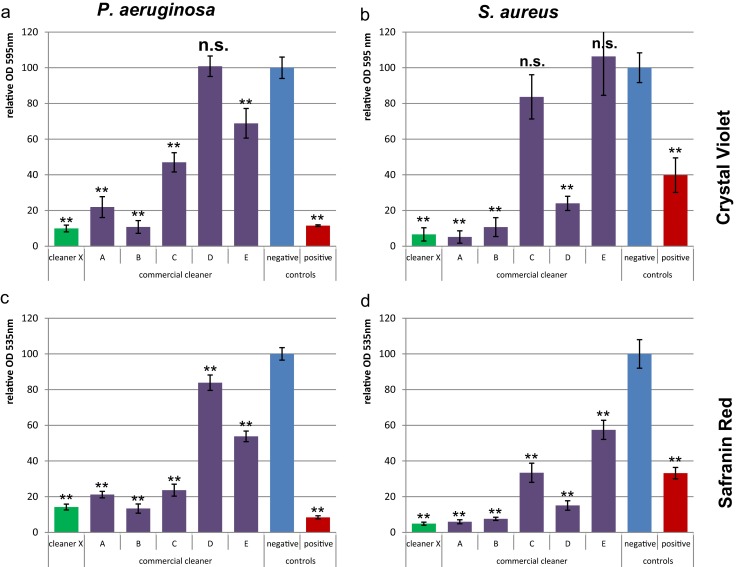
Total biomass quantification. *P. aeruginosa* (**a**, **c**) and *S. aureus* (**b**, **d**) biofilms were treated with different cleaners. The amount of biofilm was quantified by Crystal Violet (**a**, **b**) and Safranin Red (**c**, **d**) staining. The *Y*-*axis* represents the biofilm amount relative to the negative control. *Error bars* are generated from six replicas. A *t* test was applied to each cleaner treatment compared to the negative control to calculate if the differences are statistically highly significant (*two asterisks*, *p* < 0.001) or not significant (*n.s.*, *p* > 0.05)

Without cleaner treatment (negative control), Crystal Violet staining resulted in an absolute absorbance value of 1.0 for *P. aeruginosa* and 0.3 for *S. aureus*, which can be easily and reliably detected (Fig. 1a, b). The standard deviations were generally less than 5 %. However, a relatively high amount of biomass was required to allow distinction from the background. The staining background of the plain plate had approximately a signal of 0.05 (Table 1). This allows reliable detection of up to 98.7 % reduction for *P. aeruginosa*. For *S. aureus* with an absolute signal of 0.3 for the negative control, the MDR value is about 83 %.

Safranin Red staining (Fig. 1c, d) led to similar relative standard deviations but lower absolute absorbance values compared to Crystal Violet. This complicates the differentiation from the background and results in higher detection limits of maximal 97.1 % reduction of *P. aeruginosa* and 92.9 % reduction of *S. aureus*.

Congo Red staining was also tested for biomass quantification, but the absolute absorbance values were too low compared to those of background staining.

The methods of Crystal Violet and Safranin Red led to the same conclusion regarding the effectiveness of the tested cleaners: with cleaner X among the best while cleaners C, D, and E did not remove biofilm efficiently (Fig. 1). In both detection methods, the cleaner treatment did not exhibit an effect on the background staining of the wells without bacteria.

#### Total amount of bacterial cells

The detection of *P. aeruginosa* cells by SYTO9 (Fig. S3a) did not work sufficiently due to stronger staining of dead cells as described previously (Stiefel et al. 2015). For the detection of *S. aureus* cells with SYTO9, the ratio of the negative control signal to background noise was higher than that of Crystal Violet (Fig. S3b). According to the MDR value, SYTO9 allowed detection of 99.8 % reduction. However, standard deviations of the wells with bacteria were rather high and reproducibility was low.

**Fig. 2:**
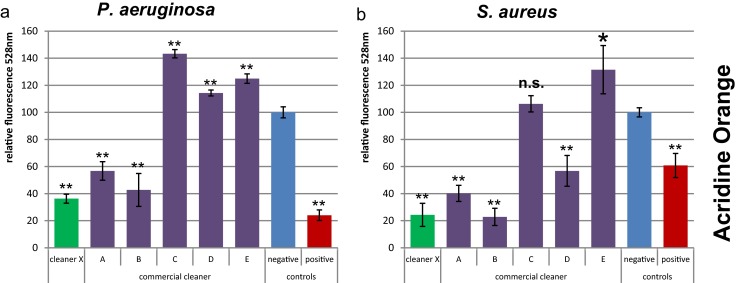
Total cell quantification by Acridine Orange staining. *P. aeruginosa* (**a**) and *S. aureus* (**b**) biofilms were treated with different cleaners. The *Y-axis* represents the fluorescent signal values relative to the negative control. *Error bars* are generated from six replicas. A *t* test was applied to each cleaner treatment compared to the negative control to calculate if the differences are statistically significant (*one asterisk*, *p* < 0.05), highly significant (*two asterisks*, *p* < 0.001), or not significant (*n.s.*, *p* > 0.05)

For Acridine Orange, the effect of stronger staining of dead cells seemed not to occur and the standard deviations were lower than that for SYTO9 staining (Fig. 2). However, cells treated with cleaners in general appeared to be stained stronger by Acridine Orange in comparison to cells in control samples.

The results of the SYTO9 and Acridine Orange staining indicated similar differences in the effectiveness of removing bacteria between the tested detergents. Cleaner X was among the best while cleaners C, D, and E did not remove bacteria efficiently (Fig. 2). Cleaner treatment did not have an influence on the binding of the dyes to the plate material as no differences in background fluorescence were observed.

#### Amount of living bacterial cells

**Fig. 3:**
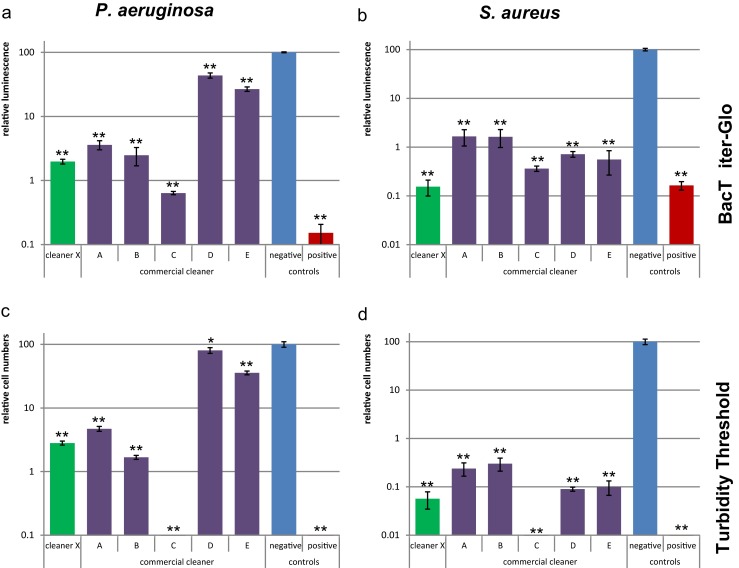
Quantification of viable cells. *P. aeruginosa* (**a**, **c**) and *S. aureus* (**b**, **d**) biofilms were treated with different cleaners. The viable cells were quantified by ATP detection with BacTiter-Glo reagent (**a**, **b**) and regrowth in the Turbidity Threshold method (**c**, **d**). The *Y-axis* represents the cell numbers relative to the negative control. *Error bars* are generated from six replicas. A *t* test was applied to each cleaner treatment compared to the negative control to calculate if the differences are statistically significant (*one asterisk*, *p* < 0.05) or highly significant (*two asterisks*, *p* < 0.001)

ATP, which is only produced and retained in living cells, was quantified by BacTiter-Glo™ Microbial Cell Viability Assay (Fig. 3a, b). This method was very sensitive and displayed a broad linear range. The luminescence signal of the negative control was about 1000 times higher than that of the background of the BacTiter-Glo solution without cells, which allows detection of up to 3 Log reductions (99.92 % for *P. aeruginosa* and 99.95 % for *S. aureus*). However, the required materials (e.g., luciferase and luciferin) are expensive, especially for analysis of large numbers of samples.

A cost-efficient alternative to ATP quantification is the turbidity threshold method, in which growth medium is added to the cells after cleaner treatment to measure the time needed to reach a certain optical density (OD) (Fig. S4). The number of remaining viable cells was obtained based on a standard curve (Fig. 3c, d). This method displayed a broad linear range and therefore is very useful for samples with large differences. However, small differences might be difficult to detect because fast-proliferating bacteria result in slight difference of a few minutes in growth. Another disadvantage is the rather long measuring time during which the plate reader is occupied.

A compromise between cost-efficiency and throughput is represented by the use of a tetrazolium salt. Iodonitrotetrazolium (INT) was selected in this study as it forms crystals that stick to the biofilm when converted to formazan. The red-violet formazan crystals were dissolved in DMSO and quantified by absorbance (Fig. S5). In the wells without cells, no formazan was formed and therefore the optical density was not increased in background wells, but the absolute optical density signal for cells treated with the negative control (0.9 % NaCl) was rather low at only 0.5, which enables the detection of up to about 2 Log reduction (99.2 % for *P. aeruginosa* and 99.5 % for *S. aureus*). With higher standard deviation and lower MDR, this method was not as precise and sensitive as the ATP quantification.

Another often used method in viability test is the staining of cells with the fluorescent dyes SYTO9 and propidium iodide (PI), resulting in green fluorescent staining of live cells and red fluorescent staining of dead cells. In the current study, it was observed that these dyes enabled the quantification of live/dead cells of biofilm in 96-well plates (Fig. S6). One advantage is that total cell and viable cell numbers could be quantified in the same plate by staining first with SYTO9 and subsequently counterstaining with PI. However, as described previously (Stiefel et al. 2015), having the problems with staining gram-negative strains, this method is not always precise for biofilm quantification in 96-well plates. For example, with SYTO9/PI staining, the biofilm treated with cleaner C exhibited higher living cell numbers than those with ATP or turbidity assays. This is likely caused by incomplete replacement of SYTO9 by PI in dead cells, as observed previously (Stiefel et al. 2015), leading to a fallaciously higher number of live cells.

The methods exploited in this work showed that cleaner X was among the best in eliminating viable cells, likely through removing the biofilm based on the results shown in Fig. 1. While all cleaners reduced viable cells of *S. aureus* effectively, cleaners A and B led to more viable cells than the other cleaners. Cleaners D and E did not reduce viable cells of *P. aeruginosa* sufficiently. Very few viable cells were detected after treatment with cleaner C, but an increased background OD at 600 nm in the turbidity method (Fig. S4a) was observed, indicating that this product contains disinfectant and many dead bacterial cells remain in the well. Similar conclusions regarding the effectiveness of the tested cleaners can be drawn from the ATP assay, turbidity threshold method, and tetrazolium salt method, but not from SYTO9/PI staining.

#### Biofilm EPS - amount of proteins

As described previously (Ahmed 2005), many standard protein quantification methods such as the Lowry and BCA assays did not work properly in the complex environment of biofilm. Often, reducing agents (e.g., most sugars) and other components of the biofilm generate wrong signals, especially if treated with cleaners. In this study, it was found that staining methods such as CBQCA and NanoOrange were influenced by the antecedent cleaner treatment and not further investigated (data not shown). SYPRO Ruby was found to have a high affinity for the plate material (both polystyrene and polypropylene), resulting in high background (Table 2).

**Table 2:**
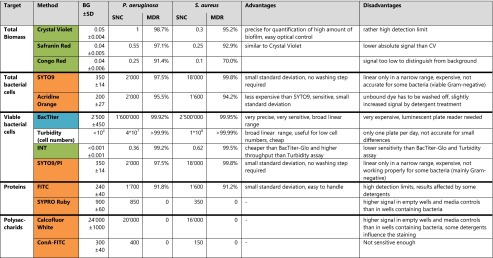
Summary and evaluation of the performance of different detection methods

Biofilm formed at 33 °C and 40 rpm for 24 h was quantified by the different methods as described in Materials and methods. Signals are measured by absorbance (green), fluorescence (orange), or luminescence (blue). Absolute signals of biofilm treated with 0.9 % NaCl (SNC - negative control) are compared with the background signal from wells without bacteria (BG) and the standard deviation of the background (SD). The *maximal detectable reduction* (MDR) value reflects the detection limits which should be distinguishable from noise of BG

**Fig. 4:**
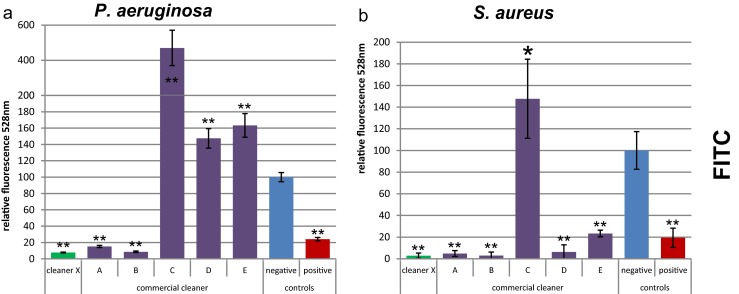
Quantification of biofilm protein by FITC staining. *P. aeruginosa* (**a**) and *S. aureus* (**b**) biofilms were treated with different cleaners. The *Y-axis* represents the fluorescent signal values relative to the negative control. *Error bars* are generated from six replicas. A *t* test was applied to each cleaner treatment compared to the negative control to calculate if the differences are statistically significant (*one asterisk*, *p* < 0.05) or highly significant (*two asterisks*, *p* < 0.001)

A simple but promising method is the staining with fluorescein isothiocyanate (FITC) (Fig. 4). The background staining of the wells without cells was rather high, but not influenced by the cleaner treatment. The signal of the negative control was only seven times higher than the background staining, and standard deviations of the background were rather high, resulting in a MDR value of 91.8 % for *P. aeruginosa* and 91.2 % for *S. aureus*. However, the standard deviations for low values were small and should allow detection of up to 95 % reduction. The quantified protein level for some cleaners (e.g., cleaner C) was too high compared to that of the negative control. This might be explained by the possibility that the detergents modified the proteins, which consequently influenced protein binding to FITC. This effect is unlikely caused by residual detergents on the plate as the background staining in empty wells was not affected.

The results of the FITC staining demonstrate that cleaner X was among the best in removing EPS proteins, while cleaners C, D, and E did not remove proteins effectively. These results led to the same conclusion regarding the effectiveness of the tested cleaners as those with methods targeting biomass and total bacteria, indicating that the method is suitable for EPS protein quantification in the 96-well plate system.

#### Biofilm EPS - amount of polysaccharides

ConA-FITC and Calcofluor White showed a high affinity toward the plate material (both polystyrene and polypropylene) resulting in high background, which makes it difficult to distinguish the biofilm signal from the background signal (Table 2). Other methods often used for sugar quantification, such as the phenol-sulfuric acid method (Dubois et al. 1951), were not sensitive enough to quantify polysaccharides of biofilm in the microplate system and not further investigated (data not shown).

## Discussion

Different methods were applied and compared, using biofilm removal with cleaners as a case study for biofilm quantification. Methods detecting total biomass such as Crystal Violet reveal how much biofilm is present. Approaches for viability detection such as the BacTiter-Glo assay and turbidity threshold method indicate how many living bacterial cells are remaining. Only when the methods for total biofilm assay and viability assay are combined can it be revealed if bacteria are removed or only killed. Quantification methods for EPS components give an insight in how much of the biofilm matrix is removed and thereby reveal the removal of the enzyme targets. However, such methods are difficult to perform in 96-well plates due to low sensitivity or high background. A screening for different fluorescently labeled lectins could help to identify more sensitive staining of polysaccharide. Taking into account that most protein and polysaccharide quantification methods are not able to differentiate between EPS and cell membrane compounds such as peptidoglycan and LPS, it is anyway difficult to quantify the EPS alone. Nevertheless, with the knowledge of total biomass in comparison with bacteria quantification, estimations can be made on the amount of EPS.

In the case of biofilm cleaning, the most important parameters for cleaning efficiency are total biomass and living bacterial cells. The presence of viable cells will enable fast recolonization if enough nutrients are available. When an inefficient cleaner with disinfection properties is used, nutrients could be derived from dead bacterial cells and EPS and further used for bacterial adhesion and proliferation (Finkel and Kolter 2001). The different methods used here allowed detailed investigation of the cleaners regarding their killing and removing performance. It was observed that cleaner C did not remove *S. aureus* biofilm based on CV and Acridine Orange staining (Figs. 1b and 2b), but led to almost no viable cells detected by the BacTiter-Glo assay and turbidity method (Fig. 3b, d). Only the results of both total biomass and viability assays allowed the conclusion that cleaner C rather killed and fixed bacterial cells than removing the biofilm.

The performance of different cleaners in biofilm removal is summarized in Table 1. Cleaners A and B removed bacteria and EPS of both species efficiently, and did not kill the bacteria. Cleaner C partially removed *P. aeruginosa* but did not remove *S. aureus*. However, it killed both species effectively. Cleaner D partially removed *S. aureus* but neither removed nor killed *P. aeruginosa*. Cleaner E did not remove any of the two species but killed *S. aureus*. The novel cleaner X efficiently removed biofilm of both species and was only slightly biocidal.

In this study, 13 different assays which target different parts of biofilm were conducted and compared in a microplate model. The advantages and disadvantages of the tested methods are summarized in Table 2. It can be concluded that different methods are required to determine if a cleaner kills or removes biofilm. Depending on the target, it is important to choose the correct quantification method. For disinfectants, it is necessary to quantify cell viability. However, if removal of biofilm is the objective, methods which quantify total biomass or specific compounds of the biofilm should be applied. The combination of BacTiter-Glo assay and CV staining was found to be particularly suitable to investigate if a product rather acts as a disinfectant or cleaner.

## Electronic supplementary material

ESM 1 (PDF 2.63 mb)
