# Supplementary material for: Is biofilm removal properly assessed? Comparison of different quantification methods in a 96-well plate system
Source: Appl Microbiol Biotechnol. 2016 Feb 29;100:4135–45. doi: 10.1007/s00253-016-7396-9 (PMC4824840; doi:10.1007/s00253-016-7396-9)
Supplement: Supplementary file 1 — (PDF 2.63 mb) [file 253_2016_7396_MOESM1_ESM.pdf]

Applied Microbiology and Biotechnology

Is biofilm removal properly assessed? Comparison of different quantification methods in a 96-well plate system

Philipp Stiefel[1], Urs Rosenberg[2], Jana Schneider[1], Stefan Mauerhofer[2], Katharina Maniura-Weber[1], Qun Ren[1]*

[1] Laboratory for Biointerfaces, Empa, Swiss Federal Laboratories for Materials Science and Technology, Lerchenfeldstrasse 5, CH-9014 St. Gallen, Switzerland

[2] Borer Chemie AG, Gewerbestrasse 13, 4528 Zuchwil, Switzerland

* Corresponding author
E-mail: qun.ren@empa.ch, phone: +41 58 765 7688, fax: +41 58 765 7499

Table S1: Summary of biofilm removal capacity of the cleaners tested in this study.

Cleaner	Enzymes supplemented in the cleaners	Claim for Biofilm removal	Biomass (CV) (SR)		Bacteria (AO) (SYTO9)		Viability (BacTiter) (Turbidity)		EPS (FITC)	
			P.a.	***S.a.***	***P.a.***	***S.a.***	***P.a.***	***S.a.***	***P.a.***	***S.a.***
**A**	protease, lipase, amylase	yes	++<br>78%<br>79%	+++<br>95%<br>94%	++<br>43%<br>84%	+++<br>60%<br>99%	+++<br>96.4%<br>95.3%	++<br>98.3%<br>99.8%	++<br>85%	+++<br>95%
**B**	protease, amylase, cellu-lase	yes	+++<br>89%<br>87%	+++<br>89%<br>92%	+++<br>57%<br>92%	+++<br>77%<br>99%	+++<br>97.5%<br>98.3%	++<br>98.4%<br>99.7%	+++<br>91%	+++<br>97%
**C**	protease, lipase, amylase	none	++<br>53%<br>76%	+<br>16%<br>67%	0<br>-%<br>-%	0<br>-6%<br>51%	+++<br>99.4%<br>100%	+++<br>99.6%<br>100%	0<br>-%	0<br>-%
**D**	protease, lipase, amyl-ase, cellulase, man-nanase	yes	0<br>0%<br>16%	++<br>75%<br>84%	0<br>-%<br>-%	++<br>43%<br>88%	+<br>56%<br>20%	+++<br>99.3%<br>99.9%	0<br>-%	+++<br>95%
**E**	none	yes	+<br>31%<br>46%	0<br>0%<br>43%	0<br>-%<br>-%	0<br>-32%<br>50%	++<br>73%<br>64%	+++<br>99.4%<br>99.9%	0<br>-%	++<br>77%
**X**	4 enzymes	yes	+++<br>90%<br>86%	+++<br>93%<br>95%	+++<br>64%<br>90%	+++<br>76%<br>99%	+++<br>98.0%<br>97.2%	+++<br>99.8%<br>99.9%	+++<br>92%	+++<br>97%

% reduction measured by different methods is indicated (order as indicated in the heading).

+++: biofilm reduction >80% in average of the used methods or >90% in one of the method;

++: biofilm reduction >50% in average, but none >90%;

+: biofilm reduction 25% - 50% in average and

0: biofilm reduction <25% in average.

Those terms were applied to all methods, except that for the viability of *S.a.* the threshold for '+++' was set to 99% due to strong reduction of all cleaners.

P.a.: *Pseudomonas aeruginosa*; *S.a.*: *Staphylococcus aureus*;

CV: Crystal Violet staining

SR: Safranin Red staining

AO: Acridine Orange staining

SYTO9: SYTO9 staining

BacTiter: BacTiter-Glo assay

Turbidity: Turbidity Threshold method

FITC: Fluorescein isothiocyanate

Figure S1: Arrangement of the samples in 96-well plates during biofilm formation (a), cleaner treatment (b) and staining (c).

Figure S2: Location of biofilm. *P. aeruginosa* (a) and *S. aureus* (b) biofilms were formed for 24 hours at 33°C, and subsequently stained with Crystal Violet for visualization. The locations with most biofilm are indicated by arrows.

Figure S3: Quantification of total cell content by SYTO9. *P. aeruginosa* (a) and *S. aureus* (b) biofilms were treated with different cleaners. Y-axis represents the fluorescent signal values relative to the negative control. Error bars are generated from six replicas. A t-test was applied to each cleaner treatment compared to negative control to calculate if the differences are statistically significant (*, $p<0.05$) or highly significant (**, $p<0.001$).

Figure S4: Quantification of viable cells by proliferation for the Turbidity Threshold method. *P. aeruginosa* (a) and *S. aureus* (b) biofilms were treated with different cleaners. Optical density of growing cells after the treatment is displayed over time. Mean value of 6 replicas is shown.

Figure S5: Quantification of viable cells by Tetrazolium salt. *P. aeruginosa* (a) and *S. aureus* (b) biofilms were treated with different cleaners. Y-axis represents the OD values relative to the negative control. Error bars are generated from six replicas. A t-test was applied to each cleaner treatment compared to negative control to calculate if the differences are statistically highly significant (**, $p<0.001$).

Figure S6: Quantification of viable cells by SYTO9/PI staining. *P. aeruginosa* (a) and *S. aureus* (b) biofilms were treated with different cleaners. Y-axis represents the fluorescent signal values relative to the negative control. Error bars are generated from six replicas. A t-test was applied to each cleaner treatment compared to negative control to calculate if the differences are statistically highly significant (**, $p<0.001$) or not significant (n.s., $p>0.05$).

Fig. S1

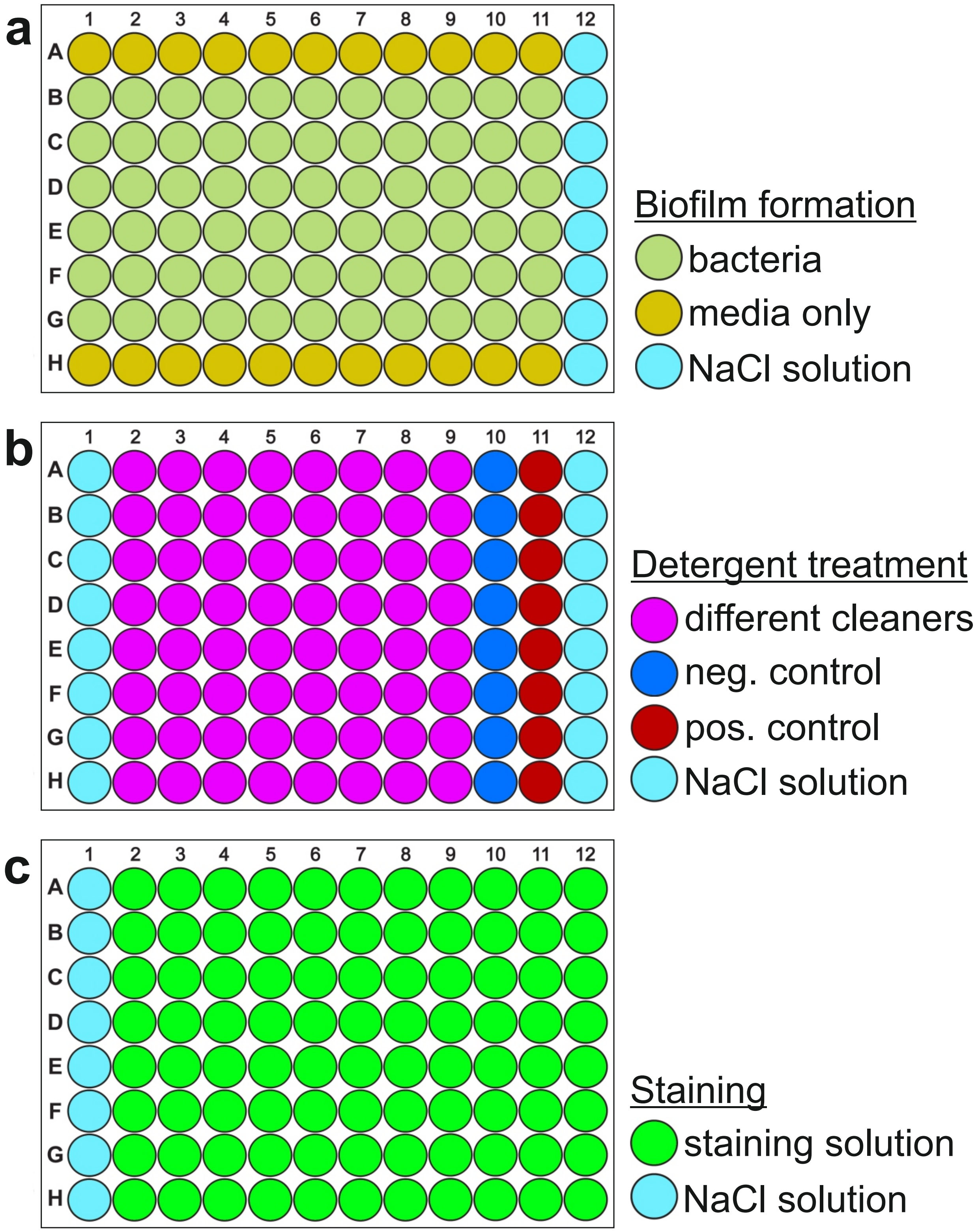
a
1 2 3 4 5 6 7 8 9 10 11 12
A B C D E F G H
Biofilm formation
bacteria
media only
NaCl solution
b
1 2 3 4 5 6 7 8 9 10 11 12
A B C D E F G H
Detergent treatment
different cleaners
neg. control
pos. control
NaCl solution
c
1 2 3 4 5 6 7 8 9 10 11 12
A B C D E F G H
Staining
staining solution
NaCl solution

Fig. S2

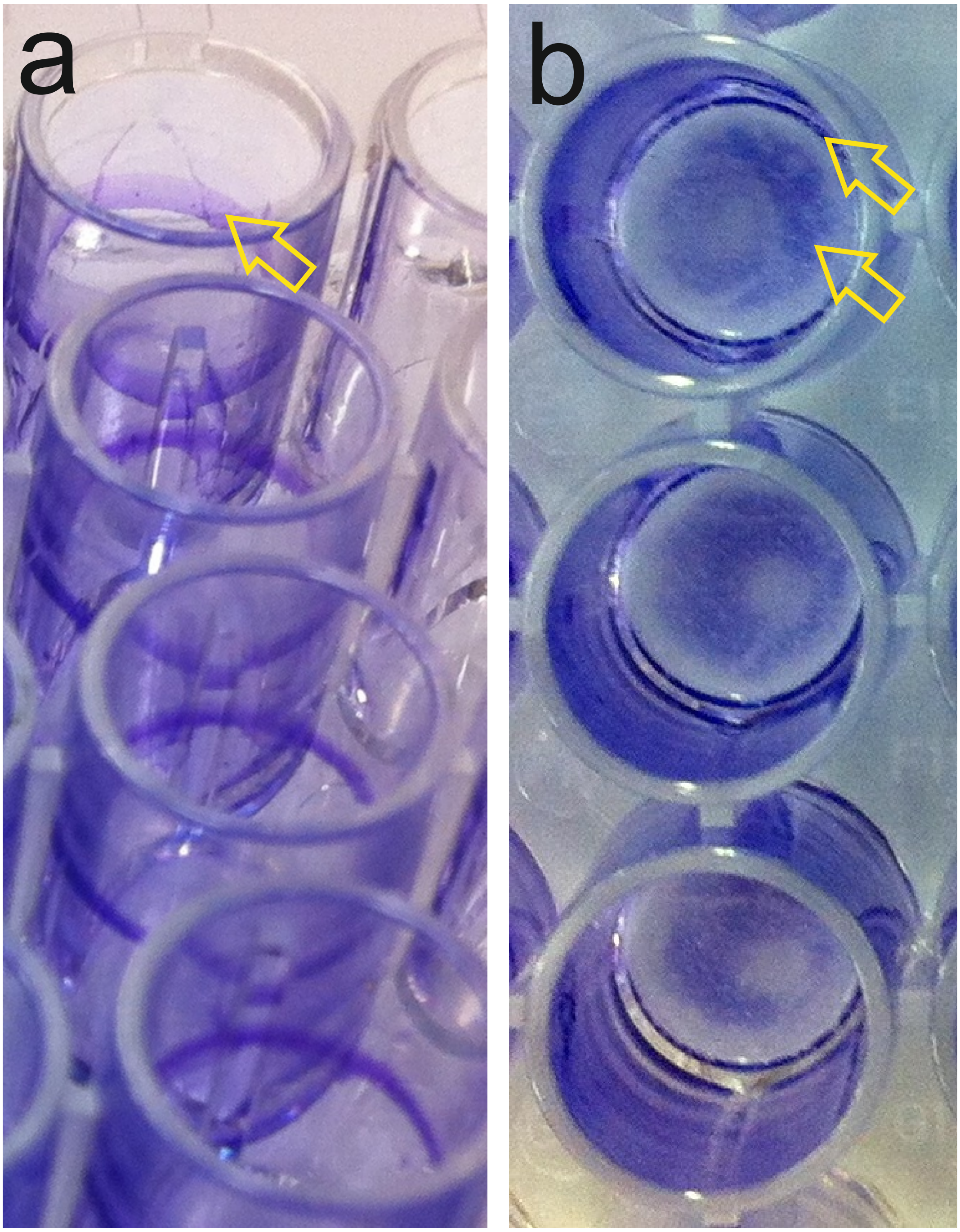

Fig. S3

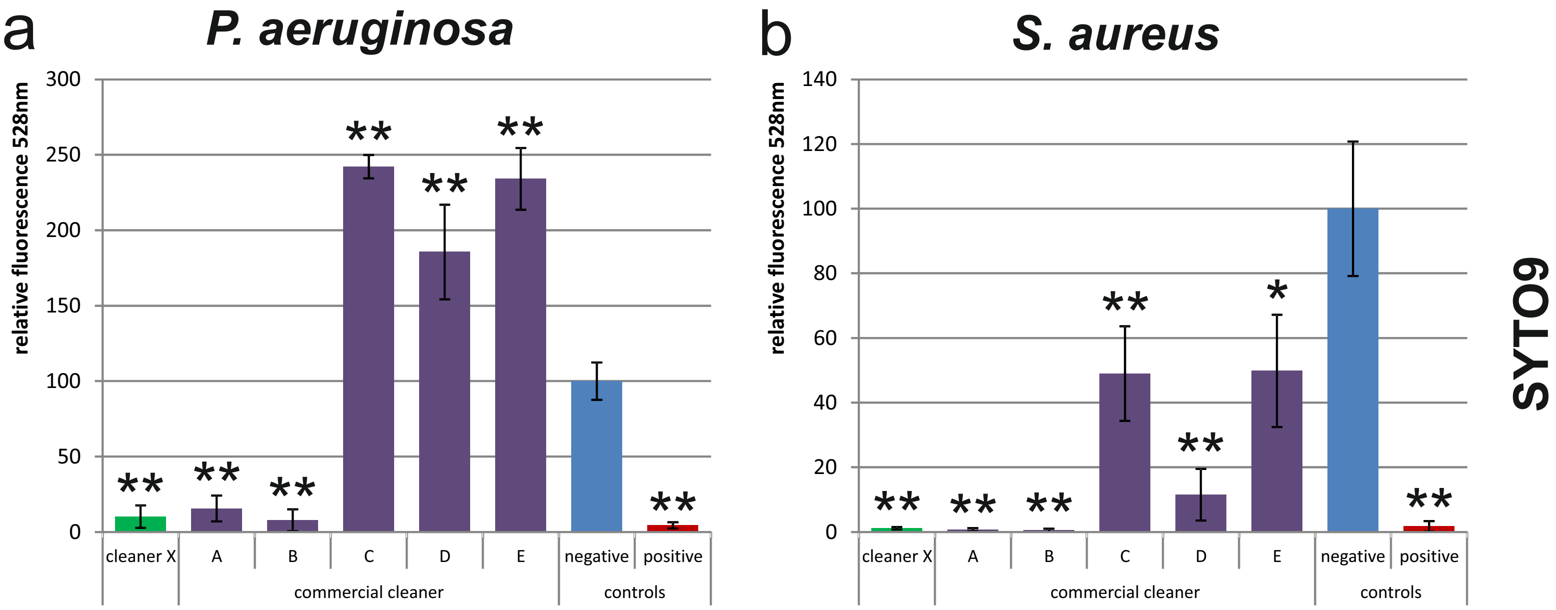
a
P. aeruginosa
relative fluorescence 528nm
300
250
200
150
100
50
0
**
**
**
**
**
**
**
cleaner X
A
B
C
D
E
negative
positive
commercial cleaner
controls
b
S. aureus
relative fluorescence 528nm
140
120
100
80
60
40
20
0
**
**
**
**
**
*
**
cleaner X
A
B
C
D
E
negative
positive
commercial cleaner
controls
SYTO9

Fig. S4

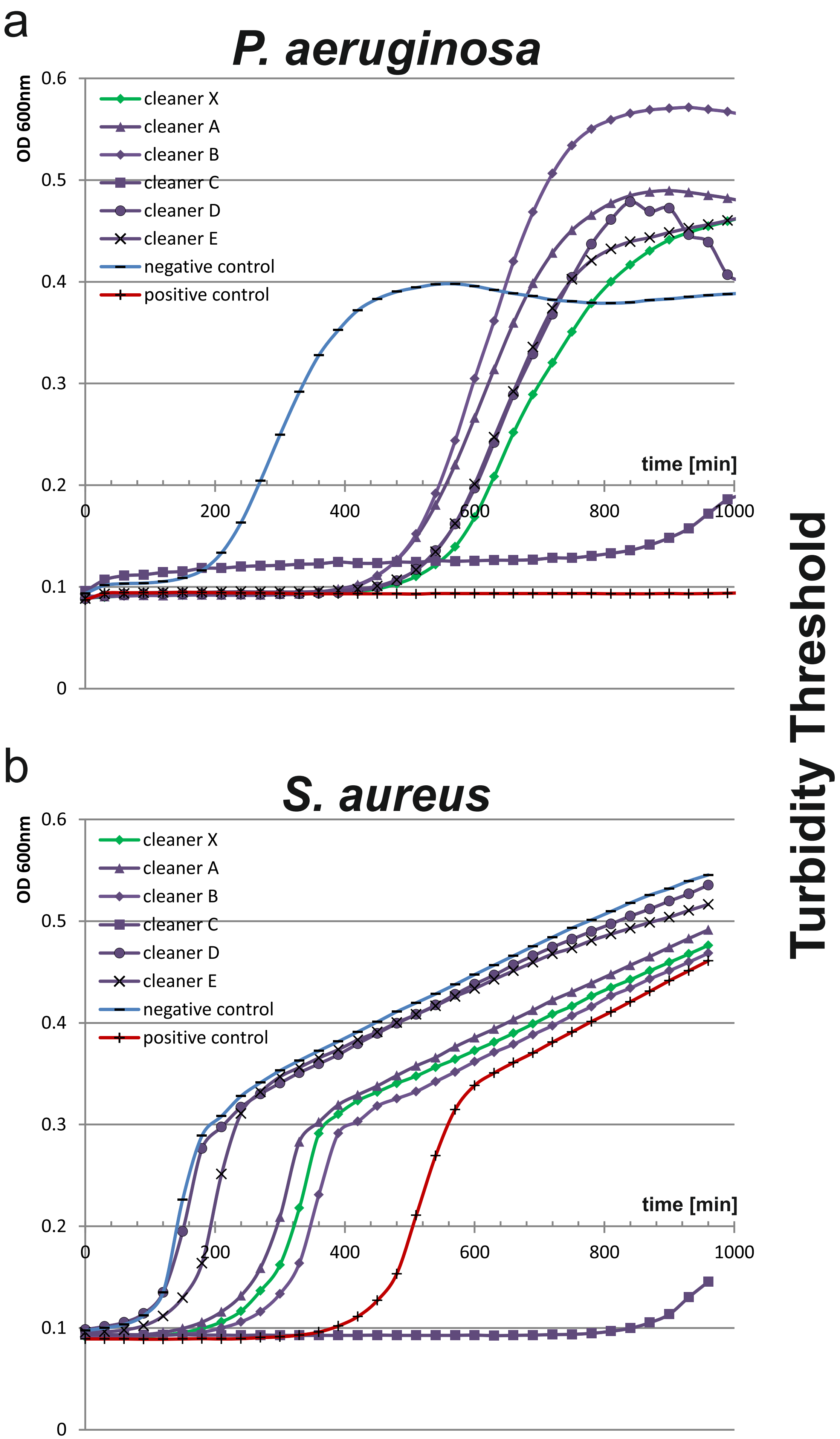
a
P. aeruginosa
OD 600nm
0.6
0.5
0.4
0.3
0.2
0.1
0
time [min]
0
200
400
600
800
1000
cleaner X
cleaner A
cleaner B
cleaner C
cleaner D
cleaner E
negative control
positive control
b
S. aureus
OD 600nm
0.6
0.5
0.4
0.3
0.2
0.1
0
time [min]
0
200
400
600
800
1000
cleaner X
cleaner A
cleaner B
cleaner C
cleaner D
cleaner E
negative control
positive control
Turbidity Threshold

Fig. S5

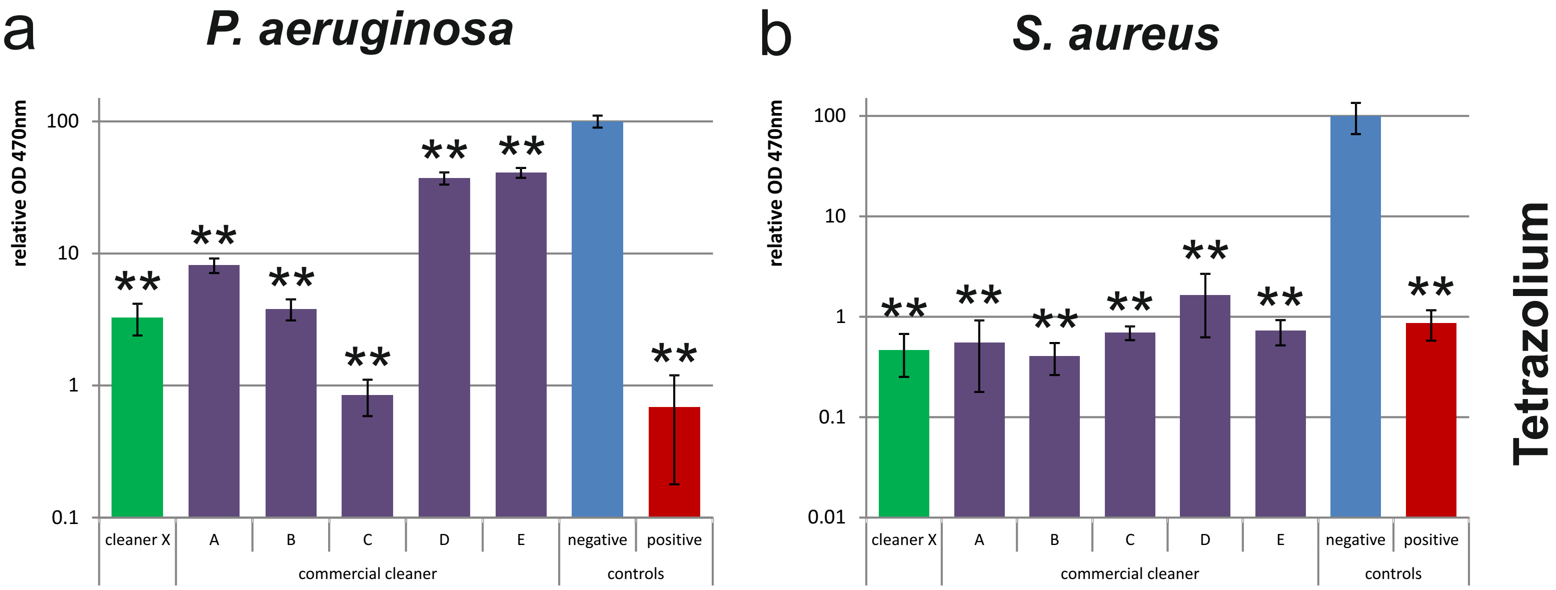

a
P. aeruginosa
relative OD 470nm
100
10
1
0.1
**
cleaner X
A
B
C
D
E
negative
positive
commercial cleaner
controls
b
S. aureus
relative OD 470nm
100
10
1
0.1
0.01
cleaner X
A
B
C
D
E
negative
positive
commercial cleaner
controls
Tetrazolium

Fig. S6

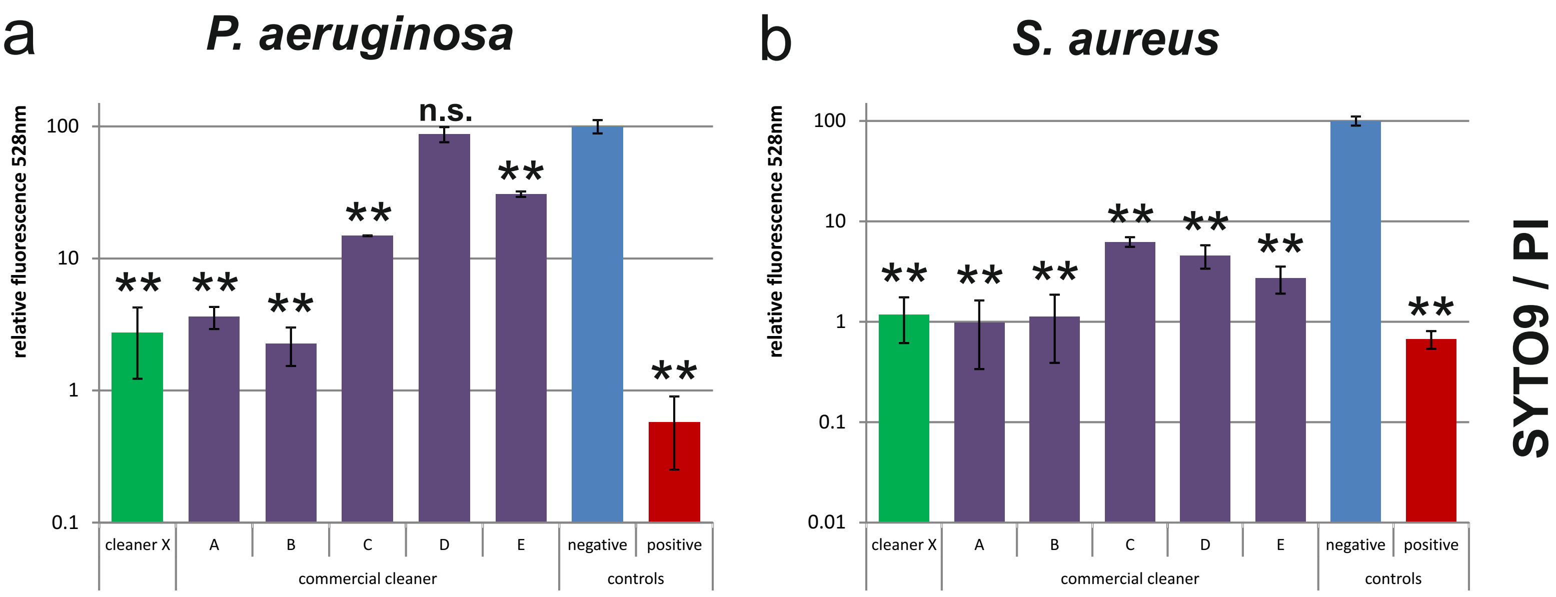

a
P. aeruginosa
relative fluorescence 528nm
100
10
1
0.1
**
**
**
**
n.s.
**
**
cleaner X
A
B
C
D
E
negative
positive
commercial cleaner
controls
b
S. aureus
relative fluorescence 528nm
100
10
1
0.1
0.01
**
**
**
**
**
**
**
cleaner X
A
B
C
D
E
negative
positive
commercial cleaner
controls
SYTO9 / PI
